# Characterization of Adaptive-like γδ T Cells in Ugandan Infants during Primary Cytomegalovirus Infection

**DOI:** 10.3390/v13101987

**Published:** 2021-10-03

**Authors:** Jessica Tuengel, Sanya Ranchal, Alexandra Maslova, Gurpreet Aulakh, Maria Papadopoulou, Sibyl Drissler, Bing Cai, Cetare Mohsenzadeh-Green, Hugo Soudeyns, Sara Mostafavi, Peter van den Elzen, David Vermijlen, Laura Cook, Soren Gantt

**Affiliations:** 1 BC Children’s Hospital Research Institute, University of British Columbia, Vancouver, BC V5Z 4H4, Canada; jessicaharli@gmail.com (J.T.); sranchal@student.ubc.ca (S.R.); gsaulakh@alumni.ubc.ca (G.A.); caibing@bcchr.ubc.ca (B.C.); cetarehmg@gmail.com (C.M.-G.); saram@stat.ubc.ca (S.M.); pvde@mail.ubc.ca (P.v.d.E.); l.cook@unimelb.edu.au (L.C.); 2 Department of Bioinformatics, University of British Columbia, Vancouver, BC V5T 4S6, Canada; sasha113@gmail.com; 3 Department of Pharmacotherapy and Pharmaceutics, Université Libre de Bruxelles (ULB), 6041 Gosselies, Belgium; mpapadop@ulb.ac.be (M.P.); dvermijl@ulb.ac.be (D.V.); 4 Institute for Medical Immunology, Université Libre de Bruxelles (ULB), 1050 Brussels, Belgium; 5 ULB Center for Research in Immunology, Université Libre de Bruxelles (ULB), 1050 Brussels, Belgium; 6 Terry Fox Laboratory, British Columbia Cancer Agency, Vancouver, BC V5Z 1L3, Canada; sdrissler@bccrc.ca; 7 Department of Microbiology, Infectiology and Immunology, Université de Montréal, Montréal, QC H3C 3J7, Canada; hugo.soudeyns@umontreal.ca; 8 CHU Sainte-Justine Research Centre, Montréal, QC H3T 1C5, Canada; 9 Department of Microbiology and Immunology, The Peter Doherty Institute for Infection and Immunity, University of Melbourne, Melbourne, VIC 3000, Australia

**Keywords:** CMV, gamma delta T cell, gammadelta, Vδ1, Vδ3, Vγ8, Vγ9^neg^Vδ2, cCMV, NKG2C, immune ontogeny

## Abstract

Gamma-delta (γδ) T cells are unconventional T cells that help control cytomegalovirus (CMV) infection in adults. γδ T cells develop early in gestation, and a fetal public γδ T cell receptor (TCR) clonotype is detected in congenital CMV infections. However, age-dependent γδ T cell responses to primary CMV infection are not well-understood. Flow cytometry and TCR sequencing was used to comprehensively characterize γδ T cell responses to CMV infection in a cohort of 32 infants followed prospectively from birth. Peripheral blood γδ T cell frequencies increased during infancy, and were higher among CMV-infected infants relative to uninfected. Clustering analyses revealed associations between CMV infection and activation marker expression on adaptive-like Vδ1 and Vδ3, but not innate-like Vγ9Vδ2 γδ T cell subsets. Frequencies of NKG2C^+^CD57^+^ γδ T cells were temporally associated with the quantity of CMV shed in saliva by infants with primary infection. The public γδ TCR clonotype was only detected in CMV-infected infants <120 days old and at lower frequencies than previously described in fetal infections. Our findings support the notion that CMV infection drives age-dependent expansions of specific γδ T cell populations, and provide insight for novel strategies to prevent CMV transmission and disease.

## 1. Introduction

Cytomegalovirus (CMV) infects most of the world’s population, beginning in early childhood [1]. Postnatal CMV infection is frequently asymptomatic and rarely causes disease in immunocompetent individuals; however, population-based studies have indicated an association between CMV infection and all-cause mortality [2]. Congenital CMV infection (cCMV) is a major cause of childhood deafness and other neurodevelopmental disabilities [3]. Immunological changes during early life likely play an important role in the differential pathogenesis of CMV infection acquired in utero versus following birth. Infants and young children have weaker control of CMV replication compared to adults, as evidenced by their higher level and longer duration of viral shedding in saliva and urine [4,5]. Viral shedding by young children is a major mode of CMV transmission to adults, including pregnant women, and is therefore thought to be a key driver of cCMV [6,7]. Thus, a better understanding of the immune control of viral replication and viral shedding in early life could facilitate the development of a vaccine to prevent cCMV.

γδ T cells represent only 0.5–10% of total circulating lymphocytes, and have traditionally been considered an innate subset of T cells. Instead of a heterodimer between α and β chains as in conventional T cells, the γδ T cell receptor (TCR) is formed by γ and δ chains [8], which have the capacity for gene rearrangement [9]. γδ T cells have potent anti-tumor and antiviral immune functions, and can potentially recognize a wide range of antigens independent of presentation by classical major histocompatibility complex (MHC) [10,11]. Although γδ T cells are a minor subset of T cells in peripheral blood, their frequencies are higher in other anatomical sites including mucosal tissues [12]. The ability to react rapidly once activated makes γδ T cells particularly enticing candidates for immunotherapy and vaccination [13,14].

γδ T cells have been shown to respond to CMV infection in transplant recipients [15,16] and healthy seropositive individuals [17]. Expansions of γδ T cells in peripheral blood have been found to correlate with the resolution of lytic viral replication during acute CMV infection [15], a phenomenon that is not observed following infection with other herpesviruses such as Epstein–Barr virus (EBV) or herpes simplex virus [16]. Similar to CD8^+^ αβ T cells, CMV infection induces long-lived oligoclonal CMV-specific γδ T cell populations that exhibit an effector/memory phenotype and potent antiviral cytotoxicity, largely through IFN-γ production [18,19]. Although γδ T cell responses to CMV have predominantly been studied in chronically infected adults or transplant patients, their role in responding to fetal CMV infection has also been described [20]. Of note, γδ Τ cells arise earlier in gestation than αβ T cells [21,22], suggesting that they may be particularly important in prenatal immunity.

Subpopulations of γδ T cells are classified based on which γ and δ chains they express. Vγ9Vδ2 cells are the most abundant and best-studied subset in human adult peripheral blood, are considered to be more ‘innate-like’ and have not been observed to expand or acquire specific antiviral functions in response to CMV infection [22,23,24]. Vδ1 cells and, at a lower frequency, Vδ3 cells are more tissue-associated than their Vδ2 counterparts and expand during CMV infection and are considered to be more ‘adaptive-like’ [17]. Of note, we acknowledge that the nomenclature of adaptive-like and innate-like is not currently in common use; however, we feel that these terms best communicate the messages in this paper, particularly how the kinetics of subsets are altered following CMV primary infection and resemble a CD8^+^ T cell adaptive immune response [18,19].

In cCMV, Vγ9^neg^ cells expand irrespective of pairing with the 3 delta chains (Vδ1, Vδ2 and Vδ3). However, a striking enrichment of a Vγ8Vδ1 TCR clonotype (δ1-CALGELGDDKLIF/γ8-CATWDTTGWFKIF) has been observed in congenitally infected newborns [20] and is preferentially generated by the fetal thymus [25]. This public clone is not detected in adults, indicating a developmentally dependent CMV-specific γδ T cell response in utero.

In this study, we aimed to comprehensively characterize γδ T cell populations in infants infected with CMV prenatally and during the first year of life, and compare them to uninfected infants, as well as to CMV-infected and uninfected older children and adults to determine age-related changes in γδ Τ cells and phenotypes. Furthermore, we specifically sought to determine the existence and prevalence of the public fetal clonotype in infants postnatally infected with CMV.

## 2. Materials and Methods

### 2.1. Study Population and Diagnosis of CMV Infections

**Figure 1:**
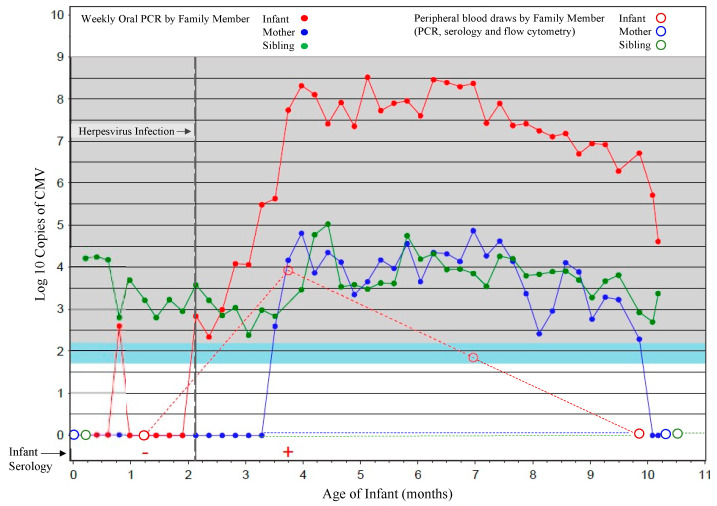
Sample scheme for cohort participants. A representative household is shown in which results for oral swabs (filled circles) collected weekly for viral PCR, and blood samples (open circles) collected at 6 weeks and then every 4 months thereafter for infants and at the time of delivery, and 1 year later for mothers and siblings, for viral PCR, serology, and flow cytometric analyses, are shown. Sampling of the infant is shown in red, mother in blue and sibling green.

Informed consent was obtained from all participants involved in the study. Human ethics approvals were obtained from the relevant research ethics boards in Kampala, Uganda (HS1181); University of Washington in Seattle, WA, USA (31713); Université Libre de Bruxelles, Belgium (P2006/151) and University of British Columbia, Vancouver, Canada (H13-01994). Pregnant women receiving prenatal care were recruited and consented in Kampala, as described previously [7,26]. Eligibility criteria for mothers included documented human immunodeficiency virus type 1 (HIV) status and family units were visited weekly and clinical information, oral swabs, and peripheral blood samples were collected from infants starting at 6 weeks of age and every 4 months thereafter, and from mothers and other children under the age of 7 years in the home at baseline (birth of infant) and 12 months later (Figure 1). All HIV-infected women and their infants received clinical care at prevention of mother-to-child HIV clinics, where follow-up and HIV testing was performed according to Ugandan National guidelines. Blood samples for the study were collected in heparin vacutainers, and plasma and peripheral blood mononuclear cells (PBMC) obtained via density gradient centrifugation over Ficoll-Paque (GE Healthcare, Chicago, IL, USA). PBMCs were subsequently cryopreserved at 1 × 10^7^ cells/mL in freezing media and plasma were stored at −80 °C.

Oral swabs and plasma samples were tested for CMV, EBV and human herpesvirus 6 (HHV6) as previously described using quantitative polymerase chain reaction (qPCR) [26,27]. Diagnoses of primary CMV infections were determined by detection of viral DNA in the plasma or oral swabs and confirmed with serology (immunoglobulin M and G enzyme-linked immunosorbent assay kits, Wampole [Alere], Boucherville, QC, Canada) [26]. If primary CMV infection criteria were met at first sampling and within the first 3 weeks of life, then infection was considered congenital (Figure 1). The cumulative incidence of postnatal CMV infection at 6 months of age was 48.2% (95% confidence interval, range 32–67.4%) and at 12 month of age 59.3% (95% confidence interval, range 42.2–77.1%) [7,26]. Viral loads in blood and oral swabs were also measured by qPCR and confirmed by serology in mothers and older children (all were confirmed to be CMV-infected). Additional blood samples from CMV-infected and -uninfected healthy immunocompetent Canadian adults (determined by IgG and IgM serological test results from the British Columbia CDC), as well as umbilical cord blood samples (assumed to be CMV-uninfected), were obtained as controls because all Ugandan mothers and children were CMV-infected. Lastly, TCR sequence data from 4 fetal and 12 cord cCMV samples from a previously described Belgian cohort and 2 CMV-uninfected cord samples were included for comparison [20].

### 2.2. Flow Cytometry

An initial immunophenotyping panel was used to characterize samples from all subjects and time points. PBMCs were thawed in a 37 °C water bath and transferred into pre-warmed (37 °C) R10 media (10% fetal bovine serum [FBS, Sigma, St. Louis, MO, USA] and 90% RPMI-1640 [Gibco, Amarillo, TX, USA]) and centrifuged at 500× *g* for 5 min to remove freezing media. PBMCs were resuspended in R10, stained with Trypan blue (Gibco) for cell enumeration and counted using a hemocytometer. Cells were washed a second time and resuspended in phosphate buffered saline (PBS, Gibco) and transferred into 96-well U-bottom plates at 1 × 10^6^ cells/well for staining. PBMCs were stained with viability dye eFluor780 (Thermo Fisher Scientific, Waltham, MA, USA; 1:1000 dilution in PBS), washed and the following monoclonal antibodies were used: CD3-V500 (clone UCH1; Becton Dickinson [BD, Franklin Lakes, NJ, USA]; 1:100), CD57-BV421 (NK-1; BD; 1:100), CD16-FITC (3G8; BD; 1:100), γδ TCR (IIF2; PE-Cy7; BD; 1:100), NKG2C-PE (no clone; R&D systems, Minneapolis, MN, USA; 1:50) and CD56-BV650 (HCD56; BioLegend, San Diego, CA, USA; 1:100). An additional biological control with known positive populations was stained and used to standardize staining between experiments and to set positive gates. The maximum number of events were acquired (range: 2.14 × 10^6^–0.48 × 10^6^ cells) on a conventional 4-laser Fortessa X20 cytometer (BD) and analyses were performed using FlowJo (v10.1, BD).

A secondary 21-color panel was subsequently designed to characterize γδ T cells using spectral cytometry (5-laser Cytek^TM^ Aurora, Cytek Biosciences, Fremont, CA, USA). A subset of Ugandan cohort subjects and time points were examined, with Canadian adults and cord blood samples as controls. PBMCs were thawed and stained with Zombie blue viability dye (BioLegend; 0.25:100), washed and stained with antibody surface markers in Brilliant Stain Buffer (BD) for 30 min. All incubations were performed in the dark at room temperature. The following antibodies were used: Vγ9-BU395 (clone B3; BD; 1:50), CD3-Pacific Blue (UCHT1; custom supplier; 1:100), CD4-BUV661 (Sk3; BD; 1:100), γδ TCR-AR700 (IF2; BD; 1:20), NKG2C-PE (no clone; R&D systems; 1:50), HLA-DR-BUV805 (Tu39; BD; 1:50), CD8- PerCP-e710 (SK1; Invitrogen, Waltham, MA, USA; 1:100), CD1d tetramer-APC (human CD1d loaded with ligand PBS-57; NIH Tetramer Core Facility 1:100), CD16-BV650 (3G8; BD; 1:50), CD161-PE-Cy5 (Beckman Coulter, Brea, CA, USA; 1:50), Ki67-BV421 (B56; BD; 1:50), Vγ8-biotin (R4.5.1; Beckman Coulter; 1:100), CX3CR1-BV711 (2A9-1; BD; 3:100), CD27-BV750 (O323; BD; 1.5:100), CD57-BV605 (QA17A04; BioLegend; 1:100), CD28-BV510 (CD28.8; BioLegend; 1:25), granzyme A-AF594 (CB9; BioLegend; 3:100), Vδ1- PE-Vio770 (REA173; BioLegend; 1:100), Vδ2- APC-Fire750 (B6; BioLegend; 1:100) and Vδ3-FITC (P11518; Beckman Coulter; 1:25).

After washing, cells were incubated with streptavidin-BV786 (BD) to conjugate with Vγ8-biotin, fixed for 30 min in 100 µL Cytofix/Cytoperm (BD) and followed by a 10-min incubation in 100 µL Perm 2 (1× dilution, BD). Cells were then stained with intracellular monoclonal antibodies (Ki67 and granzyme A) for 30 min. Cells were finally washed and resuspended in 200 µL PBS + 0.5% paraformaldehyde and transferred into micro FACS tubes.

### 2.3. Unsupervised t-SNE Clustering

Non-linear dimensionality reduction with t-distributed stochastic neighbor embedding (t-SNE) enabled the assessment of all flow cytometry parameters together, while visually reducing dimensions into a single image by clustering cells based on their expression of multiple parameters. The two-dimensional t-SNE image contains “islands” of phenotypically similar cells. However, the actual locations of these island clusters within the image only indicates similarities in marker expression. The t-SNE algorithm is a standard plugin in FlowJo v10.1. Our first step was to confirm that all individual .fcs files were compensated correctly. This was followed by a standard data-cleaning step to gate out doublets, dead cells and any signal fluctuations or acquisition irregularities over time. Samples were then gated on lymphocytes, CD3^+^ and total γδ T cells, and each channel checked for proper scaling to ensure all events were within range. The FlowJo plugin DownSample was then used to randomly select a specified number of events from each sample to reduce the total number of concatenated events to a target value between 200,000 and 400,000 to avoid overcrowding the t-SNE plot. DownSample.fcs files were then labeled with key words (CMV status and age) and concatenated into a single .fcs file. t-SNE plots were then generated in FlowJo. Five independent runs were conducted to assess consistency between results.

### 2.4. TCR Sequencing 

Sequencing of the γδ TCR repertoire was performed as previously described [28]. Briefly, RNA isolated from PBMC was reverse transcribed into complementary DNA using primers specific for the γ-chain (5′-CAAGAAGACAAAGGTATGTTCCAG-3′) and δ-chain (5′-GTAGAATTCCTTCACCAGACAAG-3′) with the SuperScript II RT enzyme (Invitrogen). Purified cDNA (AMPure XP beads, Beckman Coulter) was amplified and an index PCR with Illumina sequencing adapters was generated (Nextera XT Index Kit, Illumina, San Diego, CA, USA). High throughput sequencing (HTS) was completed on the Illumina MiSeq platform (V2 300 kit). Raw fastq file reads were aligned to GenBank derived V, D and J genes and used to build CDR3 sequences using MiXCR software (v.2.1.12) that were subsequently analyzed using VDJtools software (v.1.2.1).

### 2.5. Statistical Analyses

Determination of associations between cell population frequencies and covariates of interest were performed using a mixed methods linear regression analysis. The model accounted for repeated measures to accommodate for the cohort’s longitudinal sampling and testing for age, sex and HIV exposure in utero. Infants were grouped by age in months based on the timing of sample collection (approximately 1.5 (labelled as 2), 4, 8 and 12 months old); young children were grouped as either 1–2 years or 2–8 years; and mothers were grouped as HIV-uninfected or HIV-infected. Infants were defined as <365 days-old (older timepoints from infants were grouped within the appropriate age group), and cell proportions from populations with <50 cells were removed.

In all figures, horizontal bars represent the median; boxes extend to the 25th and 75th percentile and whiskers represent the 95th percentiles. P-values were adjusted for multiple comparisons (each cell population tested); one asterisk (*) indicates *p* < 0.05, and three (***) indicates *p* < 0.001. Statistical differences between HIV-infected and -uninfected mothers were determined using a Wilcoxon–Mann–Whitney test.

## 3. Results

### 3.1. γδ T Cell Frequencies Change with Age and Are Expanded in Infants Experiencing Primary CMV Infection

**Figure 2:**
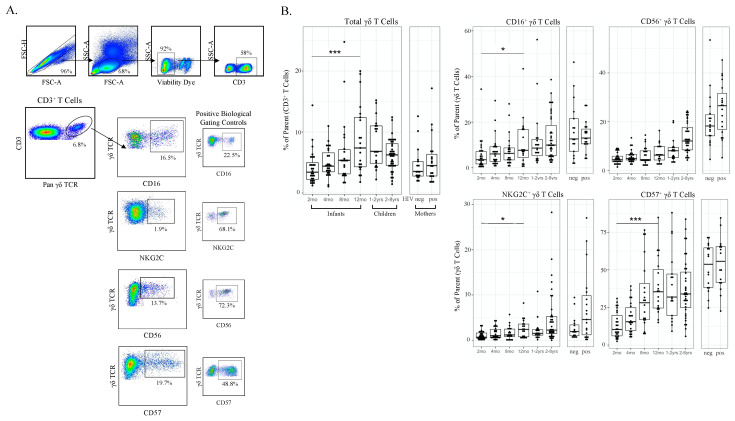

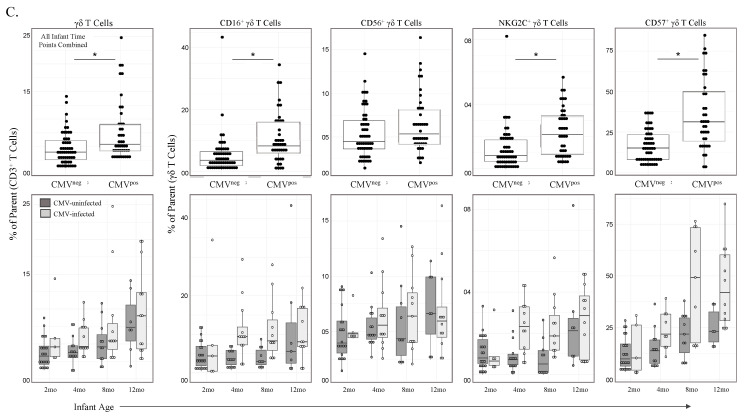
γδ T cell frequencies and phenotypic markers of activation increase with age and CMV infection. (**A**) Gating strategy for conventional flow cytometry panel. Initial flowCut cleaning in FlowJo was followed by singlet, lymphocyte, live and CD3^+^ gates as shown. γδ T cells were then gated as either CD16^+^, CD56^+^, NKG2C^+^ and CD57^+^. Positive gates were set using an internal positive biological control with known expression levels; (**B**) Total γδ T cells and CD16^+^, CD56^+^, NKG2C^+^ and CD57^+^ γδ T cells grouped by age. Infants (2, 4, 8 and 12 months of age), children (1–2 and 2–8 years old) and adult mothers were stratified by HIV serostatus (HIV-infected *n* = 17, HIV-uninfected *n* = 15); (**C**) γδ T cells from CMV-infected (*n* = 20) and CMV-uninfected infants (*n* = 8). Top row depicts cell frequencies in longitudinal samples from CMV-infected (*n* = 43) and CMV-uninfected (*n* = 57) infants (all time points) and the bottom row represents the same data plotted by both CMV infection and age in months. A mixed methods linear regression model accounting for repeated measures and testing for age, sex and HIV exposure in utero was used to determine statistical associations. Horizontal bars represent the median; boxes extend to the 25th and 75th percentile and whiskers represent the 95th percentiles. *p*-values were adjusted for multiple comparisons (each cell population tested); one asterisk (*) indicates *p* < 0.05, three (***) indicates *p* < 0.001. Statistical differences between HIV-infected and -uninfected mothers were determined using a Wilcoxon–Mann–Whitney test.

To measure differences in γδ T cell frequencies during primary CMV infection, we examined all subjects from our Ugandan longitudinal birth cohort using conventional flow cytometry (Figure 2A). Frequencies of total γδ T cells (percent of CD3^+^ T cells) in the first 12 months of life was associated with age. Furthermore, CD16, CD57 and NKG2C expression on γδ T cells were all also associated with age; CD56 expression showed a similar trend but was not statistically significant (Figure 2B). We then examined these parameters stratified by CMV infection among the 32 infants in this cohort, of whom 20 acquired CMV postnatally, 2 acquired CMV congenitally and 8 remained CMV uninfected. Compared with uninfected infants, the frequency of total γδ T cells was significantly higher in the CMV-infected infants as well as CD16^+^, CD57^+^ and NKG2C^+^ γδ T cells. CD56^+^ γδ T cells were not significantly associated with CMV infection (Figure 2C). No associations were observed between γδ T cells and EBV (Figure S1) or HHV6 (data not shown) infections. These data highlight age-related differences in γδ T cell frequencies and phenotypes associated with CMV infection in infants.

### 3.2. A Public Vδ1-CALGELGDDKLIF TCR Clonotype Can Be Detected in Post-Natal CMV Infections Occuring Very Early in Life and Rapidly Decays

**Figure 3:**
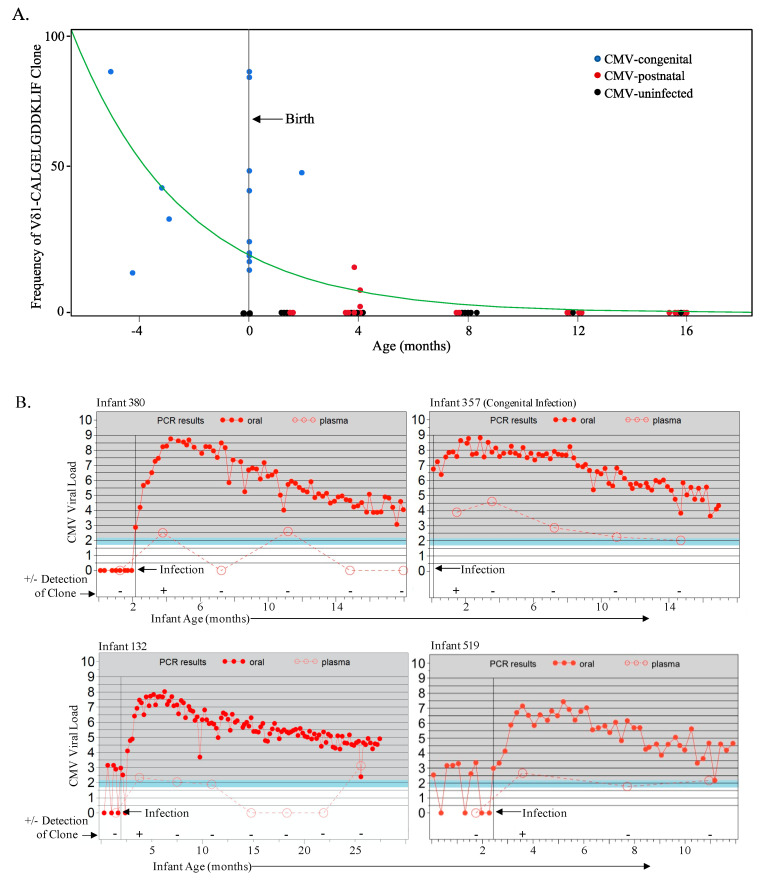
The fetal Vδ1-CALGELGDDKLIF public clone is detected in congenital and early postnatal CMV infection and decays rapidly. (**A**) Frequencies of the public Vδ1-CALGELGDDKLIF clonotype (using HTC and as a proportion of total Vδ1 cells) from the Ugandan infant cohort (17 postnatally infected, 1 congenitally infected and 8 uninfected) sampled starting from 6 weeks of age, as well as 16 Belgian cCMV samples (4 fetal and 12 cord blood) and 2 CMV-uninfected Belgian cord blood samples. Age of zero represents birth and the green spline estimates the rate of decay. Blue data points represent sampling from the congenital infections (*n* = 17), red represents samples (time points) from postnatal CMV infections (*n* = 33), and black represents samples (time points) from CMV-uninfected samples (*n* = 32); (**B**) Graphs represent data from those CMV-infected infants from the Ugandan cohort in whom the Vδ1-CALGELGDDKLIF clonotype was detected. The vertical black line denotes the age of CMV acquisition; the filled red circles indicate the CMV viral load from weekly oral swabs and the open red circles indicate the viral load in plasma as well as the time points at which PBMC were available for TCR sequencing. Every detection of the Vδ1-CALGELGDDKLIF clonotype in postnatal CMV-infected infants was from the time point immediately following infection.

Given the observed age-related differences in γδ T cell frequencies and phenotypes and associations with CMV infection in these infants, we then investigated for the presence of a public Vδ1 (Vδ1-CALGELGDDKLIF/γ8-CATWDTTGWFKIF) TCR clonotype previously observed in CMV-infected fetuses [20]. The γδ TCRs were sequenced in PBMCs from the 18 CMV-infected (1 of which was infected congenitally) and 8 uninfected infants in this cohort for whom sufficient sample was available. Using HTS, we detected the public Vδ1-CALGELGDDKLIF clonotype in four (22%) of the 18 infected infants, of whom three were postnatally infected with CMV before 120 days of age, as well as the infant with congenital infection (Figure 3A). The public clonotype was only detectable in these infants at the first time point after infection (Figure 3A,B) and was not detected in infants who became infected beyond 120 days of life (despite sustained high viral shedding) or in CMV-uninfected infants at any time point (Figure 3A). Interestingly, the public Vδ1-CALGELGDDKLIF clonotype did not persist longer than 2 months following infection at any age (Figure 3A and Figure 2B). Combining the γδ TCR sequence data from our infant cohort with those of congenitally infected pre-term and term infant cord blood and 2 CMV-uninfected cord blood samples from Belgium [20] yielded a spline trajectory that indicated an inverse correlation between frequency of Vδ1 T cells expressing the public TCR clonotype and age at infection that decayed exponentially over time (Figure 3A).

### 3.3. Adaptive-like Vδ1 and Vδ3 T Cells Have Similar Trajectories with Age and CMV Infection That Differ from the Innate-like Vδ2 T Cell Subset

Our detection of a fetal CMV-induced Vδ1 public T cell clonotype and the age-related changes in γδ T cell activity prompted the design of a large flow cytometry panel to examine frequencies of subsets with different pairings of γ and δ chains. Few studies have evaluated the prevalence of γ- and δ- T cell subsets in infancy [29,30,31] and only one has stratified these results by CMV infection [32]. Furthermore, no published flow cytometry data are available regarding Vδ3 or Vγ8 T cells during primary CMV infection, as antibodies for these targets are only available via custom order. We characterized Vδ1, Vδ2, Vδ3, Vγ8 and Vγ9 T cell subsets in a subgroup of individuals (*n* = 11 adult mothers, *n* = 15 infants, and *n* = 23 children) from our Ugandan cohort using a combination of commercial and non-commercial monoclonal antibodies. Additional cord blood (*n* = 3) and adult peripheral blood samples (*n* = 10) from healthy Canadian individuals were included as CMV-uninfected controls.

**Figure 4:**
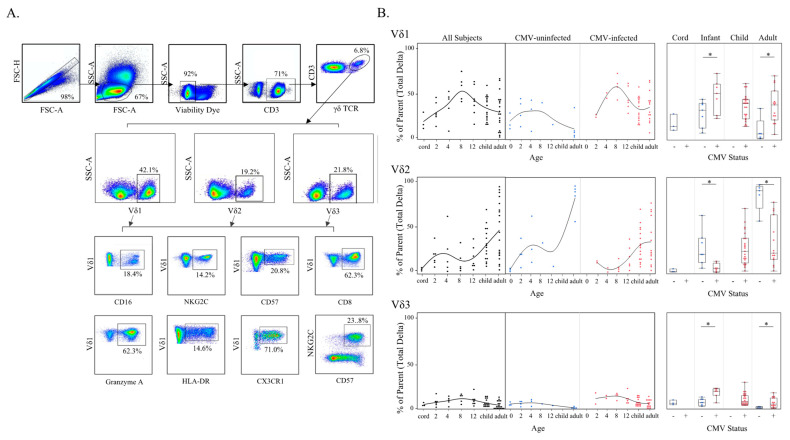
γδ T Cell δ-subsets change with age and CMV status. (**A**) Gating strategy for δ-subsets and phenotypes of γδ T cells using spectral flow cytometry. Initial flowCut cleaning in FlowJo was followed by singlet, lymphocyte, CD3^+^ and total γδ T cell gates. Next δ-subsets were gated as shown, and lastly each phenotypic marker was gated from Vδ1, Vδ2 and Vδ3 subsets (example gates are shown for Vδ1 T cells); (**B**) δ-subset frequencies (proportion of total γδ T cells) were plotted by age (left) and then additionally stratified by CMV status (center). These same data are shown on the right separated by CMV status and grouped by cord blood from Canadian births (*n* = 3), Ugandan infants (2–12 months old; *n* = 15; CMV-infected *n* = 8 and CMV-uninfected *n* = 7), Ugandan children (1–8 years old; *n* = 22) and adult subjects were Canadian (*n* = 10; CMV-infected *n* = 5 and CMV-uninfected *n* = 5) and Ugandan mothers (*n* = 11; all CMV-infected). No cord blood from congenital infections or blood samples from CMV-uninfected children older than 12 months were available for testing with this flow cytometry panel. Statistical differences shown on the right between CMV-infected and -uninfected infants and adults were determined using a Wilcoxon–Mann–Whitney test. Horizontal bars represent the median; boxes extend to the 25th and 75th percentile and whiskers represent the 95th percentiles; one asterisk (*) indicates *p* < 0.05.

Changes in Vδ1 and Vδ3 T cell subset frequencies over time among all subjects were remarkably similar yet differed from the Vδ2 T cell subset (Figure 4B). Both Vδ1 and Vδ3 T cell subsets increased from birth (cord blood) through the first year of life, and then decreased with age among older children (siblings) and adult subjects. In contrast, the Vδ2 T cell subset steadily increased in frequency with age. While both Vδ1 and Vδ3 T cells in CMV-uninfected subjects followed the same general age-related trend of increasing early in life and decreasing during the transition from childhood to adult levels, in CMV-infected individuals these subsets were significantly expanded in both infants and adults compared with CMV-uninfected individuals (Figure 4B). The trend for the Vδ2 T cell subset was the opposite, with a lower frequency in CMV-infected compared with CMV-uninfected infants and adults. Frequencies of the Vδ2 T cell subset were highest in the CMV-uninfected adults (Figure 4B).

A similar trend was detected in the Vγ compartment (Figure S2), demonstrating that the kinetics of adaptive-like γδ T cells (non-Vδ2) differ from innate-like (Vγ9Vδ2) subsets in CMV-infected infants. The frequencies of non-Vδ2 subsets followed a Gaussian trajectory reminiscent of the expansion of αβ T cell responses to primary CMV infection and their subsequent contraction as control of viral replication is gradually achieved.

### 3.4. Phenotypic Differences of γδ T Cell δ-Subsets between CMV-Infected and CMV-Uninfected Infants and Adults

**Figure 5:**
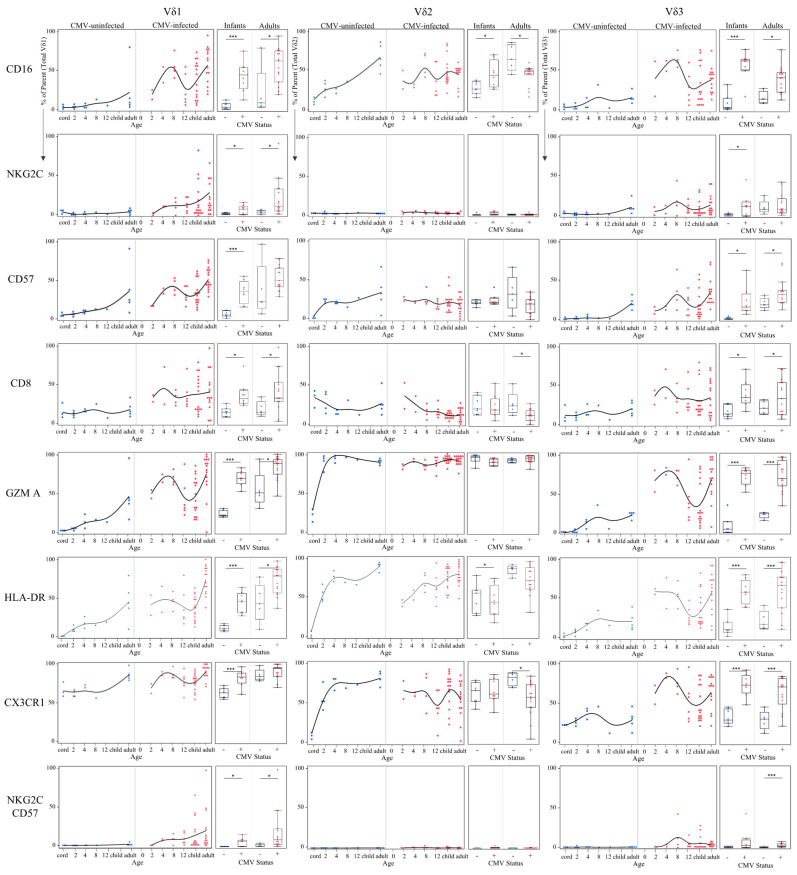
Temporal kinetics of γδ T cell δ-subsets suggest functional changes induced by CMV infection. Proportions of each phenotypic marker obtained from spectral flow cytometry were charted from either total Vδ1, Vδ2 or Vδ3 T cells were plotted by age (in months) and separated by CMV status (left plots). These same data were additionally grouped into Ugandan infants (2–12 months old; *n* = 15; CMV-infected *n* = 8 and CMV-uninfected *n* = 7) and adult subjects were Canadian (*n* = 10; CMV-infected *n* = 5 and CMV-uninfected *n* = 5) and Ugandan mothers (*n* = 11; all CMV-infected). Statistical differences between CMV-infected and uninfected infants and adults were determined using a Wilcoxon–Mann–Whitney test. Horizontal bars represent the median; boxes extend to the 25th and 75th percentile and whiskers represent the 95th percentiles; one asterisk (*) indicates *p* < 0.05, and three (***) indicates *p* < 0.001.

To further investigate age-dependent differences between the Vδ1, Vδ2 and Vδ3 γδ T cell subsets in CMV-infected and uninfected individuals, we assessed the expression levels of CD16, NKG2C, CD57, CD8, granzyme A, HLA-DR, CX3CR1, and the frequency of NKG2C^+^CD57^+^ cells, as these markers have been associated with CMV infection [17,20,23,33,34,35]. Results are shown in Figure 5 and summarized in Table 1. We found significantly higher CD16 expression on Vδ2 T cells in CMV-infected infants; however, CD16 expression was significantly lower in CMV-infected adults compared to uninfected. This differed from the Vδ1 and Vδ3 T cells, where both CMV-infected infants and adults had significantly higher expression of CD16 compared to uninfected age controls (Figure 5). Expression of the activating receptor NKG2C was almost exclusively found on Vδ1 and Vδ3 subsets in infants and adults (Figure 5). NKG2C^+^ Vδ1 T cells were significantly more frequent in CMV-infected infants and adults compared to uninfected age controls, while NKG2C^+^ Vδ3 T cells were only significantly more frequent in CMV-infected infants, not adults. A similar trend was found for CD57 (indicates an activated and differentiated phenotype), where expression on Vδ1 and Vδ3 T cells, but not Vδ2 cells, was significantly increased in CMV-infected infants and adults. CD8 alpha (α) expression has been described on γδ T cells from CMV-infected transplant patients and cCMV newborns [34,36]. We found that CD8α expression on both the Vδ1 and Vδ3 cells was increased with CMV infection in both infants and adults, and expression of CD8α was decreased on Vδ2 T cells in CMV-infected adults (Figure 5). Granzyme A is a protease found in cytotoxic granules that contributes to killing virus-infected cells [37]. Granzyme A expression in the Vδ1 and Vδ3 subsets was strongly increased with CMV infection in infants and adults, and no difference was found in expression in Vδ2 T cells between CMV infected and uninfected infants or adults (Figure 5). Similarly, HLA-DR expression on Vδ1 and Vδ3 T cells was strongly associated with CMV infection in both infants and adults, and expression by Vδ2 cells was not increased with CMV infection (Figure 5). CX3CR1 expression was increased on Vδ1 and Vδ3 cells in CMV infection in infants, and CX3CR1^+^ Vδ3 T cells were also significantly increased in CMV-infected adults. We found no increase in CX3CR1 expression on Vδ2 cells between CMV-infected and -uninfected infants and CMV infection in adults was associated with a decrease in CX3CR1 expression (Figure 5). Co-expression of CD27 and CD28 on Vδ1 and Vδ2 subsets revealed a more rapid decline in naïve γδ T cells (CD27^+^CD28^+^) in CMV-infected infants compared to uninfected infants (Figure S3). Lastly, dual expression of NKG2C and CD57 was the phenotype most associated with CMV infection as no expression was found in CMV-uninfected individuals. Interestingly, although both Vδ1 and Vδ3 T cells in CMV-infected adults and infants contained significantly more NKG2C^+^CD57^+^ cells, the temporal kinetics appeared to differ between the Vδ1 and Vδ3 T cells. NKG2C^+^CD57^+^ Vδ1 cells gradually increased with age, whereas NKG2C^+^CD57^+^ Vδ3 cells displayed a Gaussian shape, increasing until 8 months and then decreasing in frequency (Figure 5). Collectively, these data suggest a similar immunobiology for Vδ1 and Vδ3 subsets that differ from that of Vδ2 γδ T cells.

### 3.5. CMV-Associated γδ T Cell Subsets Have Unique Activated and Effector Phenotypes 

**Figure 6:**
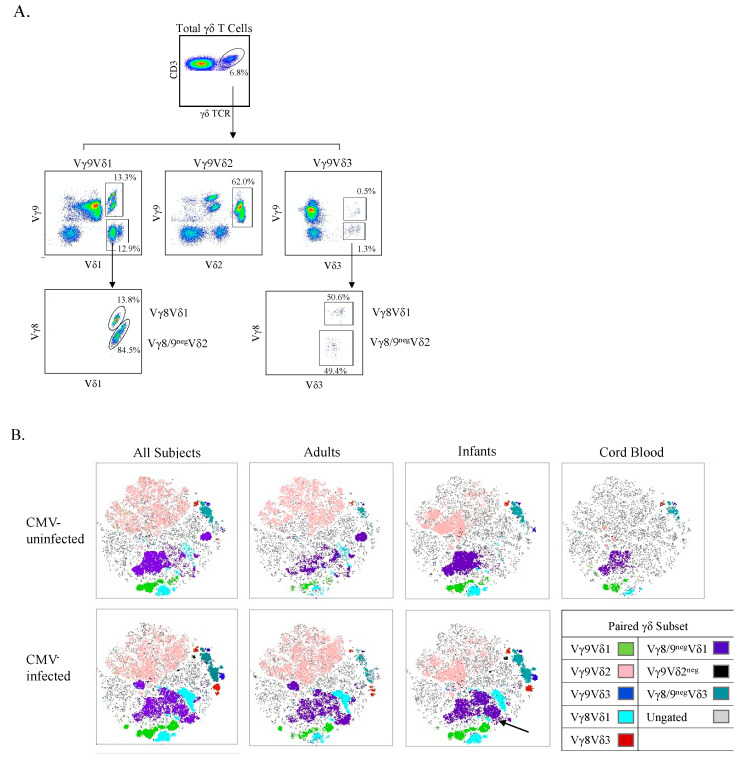
t-SNE clustering reveals specific γδ T cell populations associated with CMV infection. (**A**) Gating strategy for paired γδ T cells using spectral flow cytometry. Initial flowCut cleaning was followed by singlet, lymphocyte, CD3^+^ and total γδ T cell gates. Next paired subsets were gated as shown; (**B**) Canadian and Ugandan adults (*n* = 21), Ugandan infants (*n* = 15) and Canadian cord (*n* = 3) PBMC samples were stained, and data collected via spectral flow cytometry. Unsupervised t-SNE clusters were generated from a concatenated file of live γδ T cells from all subjects and then overlaid with manually gated paired γδ subsets. A CMV-associated CD57^+^NKG2C^+^ cluster within Vγ8^neg^/9^neg^ Vδ1 cells in infants is indicated by the black arrow.

To visualize unique clusters of cells associated with CMV infection, we used a combination of unsupervised t-SNE plots and an overlay of manually gated paired γδ T cell subsets from spectral flow cytometry (Figure 6B). Consistent with data from manual gating analyses presented above, CMV-associated γδ T cell clusters included all Vδ1 subsets (paired with Vγ8 shown in light blue, Vγ9 in green, and Vγ8^neg^/9^neg^ in purple). In infants only, we could observe Vγ9^neg^Vδ2 and Vγ9Vδ3 T cell clusters that were unique to CMV-infected individuals, shown in black and blue, respectively, in Figure 6B. The visual representation provided by the t-SNE plots illustrates that within manually gated paired γδ T cell subsets there were unique clusters that were associated with CMV infection. For example, the largest Vγ8^neg^/9^neg^Vδ1 T cell cluster (purple) appears to be naïve, given its presence in cord blood and CMV-uninfected infants; however, several Vγ8^neg^/9^neg^Vδ1 T cell clusters appear to the right of it in both CMV-infected infants and adults. This trend of subsets having two or more clusters of one γ- and δ-paired TCR subset where one of them appears only in CMV-infected subjects is apparent for all the subsets examined, except for Vγ9^neg^Vδ2 T cells (infants only) cells and the Vγ9Vδ2 T cells (shown in pink).

**Figure 7:**
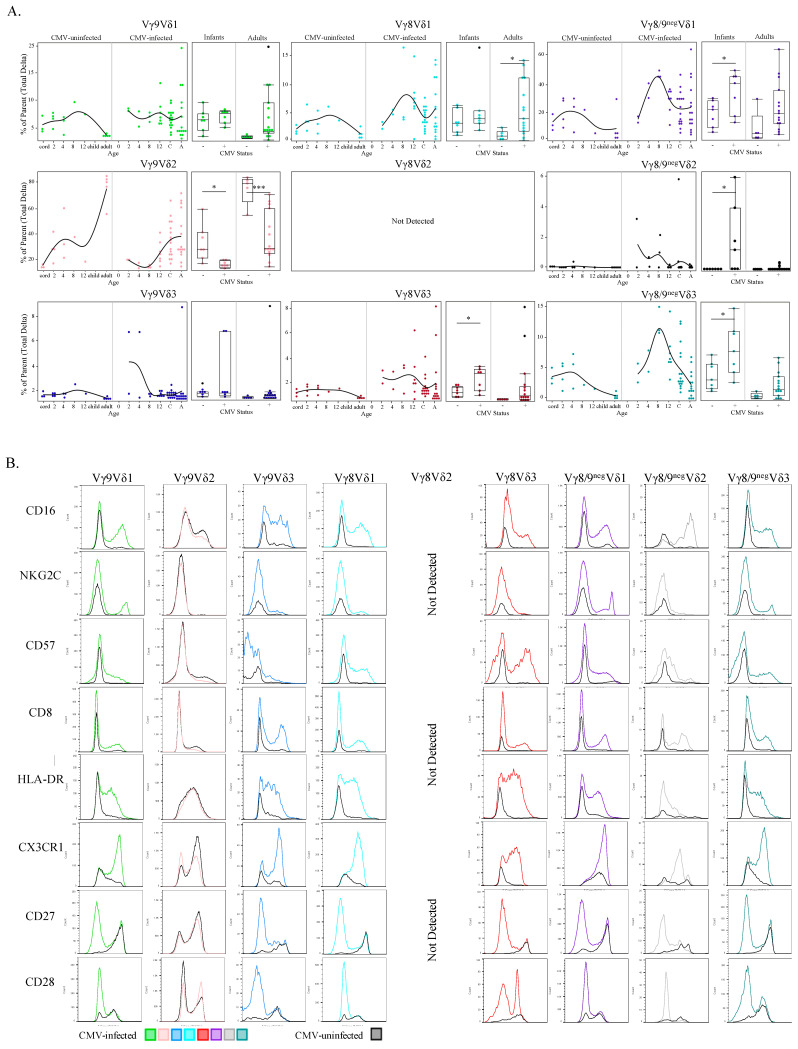
CMV-associated γδ T cell subsets show age-specific kinetics and activation phenotypes. (**A**) Proportions of each paired γδ subset (from total γδ T cells) were plotted by age and then additionally stratified by CMV status (left plots). These same data were additionally grouped into Ugandan infants (2–12 months old; *n* = 15; CMV-infected *n* = 8 and CMV-uninfected *n* = 7) and adult subjects were Canadian (*n* = 10; CMV-infected *n* = 5 and CMV-uninfected *n* = 5) and Ugandan mothers (*n* = 11; all CMV-infected); (**B**) Each row represents a phenotypic parameter and each column a paired γδ T cell subset. Histograms were generated from a concatenated file from all subjects (*n* = 39). Colored histograms represent samples from CMV-infected subjects, and grey histograms from CMV-uninfected subjects. Statistical differences between CMV-infected and -uninfected infants and adults were determined using a Wilcoxon–Mann–Whitney test. Horizontal bars represent the median; boxes extend to the 25th and 75th percentile and whiskers represent the 95th percentiles; one asterisk (*) indicates *p* < 0.05, and three (***) indicates *p* < 0.001.

No published data are available for paired (detection of γ and δ chains simultaneously) infant γδ T cell responses to CMV infection. We found that both CMV-infected infants and adults had significantly lower frequencies of the Vγ9Vδ2 population compared to their CMV-uninfected counterparts (Figure 7A). All Vδ1 T cell subsets had higher frequencies in CMV-infected subjects irrespective of age or γ-chain pairing, particularly in adult Vγ8Vδ1 T cells and infant Vγ8^neg^/9^neg^Vδ1 T cell populations. Frequencies of all Vδ3 subsets were also higher in the CMV-infected groups irrespective of age or γ pairing, yet only infant Vγ8Vδ3 and Vγ8/9^neg^Vδ3 were statistically significant. CMV-infected infants showed significantly higher frequencies of Vγ9^neg^Vδ2 T cells compared to uninfected infants. The largest frequency differences between infected and uninfected adults were in Vγ8 T cells (both Vγ8Vδ1 and Vγ8Vδ3), and for infants the Vγ9^neg^Vδ2 subset and Vδ3 T cells (both Vγ8Vδ3 and Vγ9Vδ3).

To explore the functional implications of paired γδ T cell subsets in CMV infection, expression levels of CD16, NKG2C, CD57, CD8, HLA-DR, CX3CR1, CD27 and CD28 were compared by γδ T cell subset between CMV-infected and CMV-uninfected groups (concatenated data) by generating superimposed histograms (Figure 7B). We found no difference in expression of these markers on innate-like Vγ9Vδ2 T cell subsets between infected and uninfected participants. In contrast, the other γδ T cell subsets displayed marked CMV-associated trends. Specifically, all functional activation markers tested (CD16, NKG2C, CD57, CD8, HLA-DR, and CX3CR1) were upregulated on the CMV-associated adaptive-like subsets (all subsets besides Vγ9Vδ2). Furthermore, for all subsets besides Vγ9Vδ2, there was increased CD27 and CD28 expression in the CMV-uninfected group, whereas reduced CD27 and CD28 expression was observed in CMV-infected participants, except in Vγ8Vδ3 cells that retained a population of CD28^+^ cells (Figure 7B). Of note, within CMV-infected infants there was an increase in a unique cluster of Vγ8^neg^/9^neg^ Vδ1 cells that were NKG2C^+^ CD57^+^ GranzymeA^++^ CX3CR1^++^ CD8^++^ CD16^+^ HLA-DR^+^ and CD27^neg^ CD28^neg^, this cluster is part of the purple Vγ8^neg^/9^neg^ Vδ1 cells in Figure 6B and is indicated by a black arrow.

Collectively these data show that frequencies of the innate-like Vγ9Vδ2 T cell subset were decreased in CMV-infected infants and adults, while the frequencies of all other TCR pairings were (to varying degrees) increased in CMV-infected infants and adults (compared to age matched uninfected controls). Furthermore, CMV-associated γδ T cell subsets expressed differentiated T effector phenotypes suggestive of adaptive immunobiology.

### 3.6. NKG2C^+^CD57^+^ γδ T Cell Frequency Is Associated with NKG2C Genotype and CMV Shedding In Vivo

**Figure 8:**
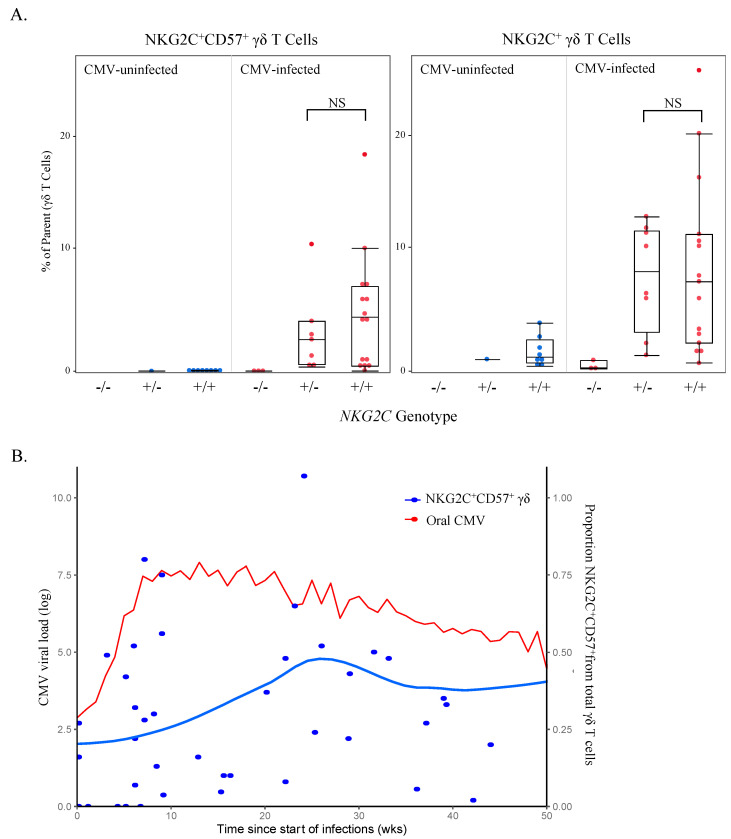
NKG2C^+^CD57^+^ γδ T cell frequency is associated with *NKG2C* genotype and oral CMV shedding. (**A**) Proportions of NKG2C^+^CD57^+^ and NKG2C^+^ γδ T cells (gated from live total γδ T cells and acquired via spectral flow cytometry as shown in Figure 4A) were grouped by genotype and CMV status (infants *n* = 9, children *n* = 8 and adults *n* = 5). No samples from CMV-uninfected individuals who were homozygous for the *NKG2C* complete deletion were available for testing; *NKG2C*−/− *n* = 0, *NKG2C*−/+ *n* = 8, *NKG2C*+/+ *n* = 24. Statistical differences between *NKG2C*−/+ and *NKG2C*+/+ genotypes in CMV-infected individuals were determined using a Wilcoxon–Mann–Whitney test. Horizontal bars represent the median; boxes extend to the 25th and 75th percentile and whiskers represent the 95th percentiles; NS indicates not significant (*p*-value > 0.05). (**B**) Weekly oral qPCR shedding (CMV log viral load) from CMV-infected Ugandan infants were concatenated and plotted from the date of primary infection. The red line indicates oral shedding in CMV-infected infants (*n* = 20), measured by qPCR. The blue line (*n* = 55) represents the mean of live γδ T cells that were CD57^+^NKG2C^+^ from the first conventional flow cytometry panel and was derived using LOESS smoothing function in R. The grey shading represents the standard error in the smoothed mean.

The evidence that NKG2C^+^CD57^+^ natural killer (NK) cell responses induced by CMV confer enhanced killing of virus-infected cells [38,39,40,41] and the association between NKG2C^+^CD57^+^ γδ T cells and CMV infection in our cohort prompted us to investigate the role of these cells in controlling viral replication in our Ugandan infant cohort. Interestingly, several groups have reported that complete *NKG2C* gene deletions are common and that *NKG2C* genotype (−/−, −/+ or +/+) appears to have a dose-dependent effect on *NKG2C* protein expression by NK cells [42,43,44]. However, *NKG2C* genotype and NKG2C protein expression have not been examined on any cell type other than NK cells. Among the Ugandan cohort participants, 20/65 (30.7%) were heterozygotes and 5/65 (7.7%) were homozygotes for the complete *NKG2C* deletion, with the remaining 40/65 having the *NKG2C+/+* genotype. As expected, we found NKG2C expression absent on NK cells in participants with *NKG2C*−/− genotypes and reduced expression in heterozygotes compared with *NKG2C+/+* individuals (Figure S2). We found a similar genotype-dependent expression of NKG2C on γδ T cells (Figure 8A); however, in contrast to NK cells, CMV-infected heterozygotes had similar levels of NKG2C^+^CD57^+^ γδ T cells compared to *NKG2C+/+* individuals, though large individual variation was evident (Figure 8A). Furthermore, when we concatenated and plotted weekly oral CMV shedding from CMV-infected infants (starting from the date of primary infection), viral load was associated with the frequencies of NKG2C^+^CD57^+^ γδ T cells (*p* = 0.00016; Figure 8B) and was not associated with NKG2C^+^ or CD57^+^ (data not shown). NKG2C^+^CD57^+^ NK cells followed a similar trend but did not have a significant association (*p* = 0.322; Figure S4). There were too few participants with *NKG2C*−/− (*n* = 1) and *NKG2C*−/+ (*n* = 3) genotypes to compare infant *NKG2C* genotypes, NKG2C^+^CD57^+^ γδ T cells and oral CMV shedding.

## 4. Discussion

Here, we show that the frequency of γδ T cells as a proportion of total T cells increased significantly over the first 12 months of life and was significantly higher in CMV-infected compared to CMV-uninfected infants. This suggests that the normal ontological expansion of γδ T cells is augmented by CMV infection during early postnatal life. We also found that CMV infection was associated with the expansion of Vγ9^neg^Vδ2 T cells in infants, as well as with the expression of activation markers by adaptive-like Vδ1 and Vδ3 γδ T cell subsets. These associations were not seen with innate-like Vδ2 γδ T cells, highlighting the specificity of these responses. Furthermore, frequencies of NKG2C^+^CD57^+^ γδ T cells were associated with infant CMV shedding during primary infection in vivo. No such associations were seen between γδ T cells and EBV or HHV6 infections. Taken together, these data suggest that CMV infection in early postnatal early life drives an expansion of specific γδ T cell populations.

Although there are few studies of γδ T cells in early postnatal life, the available data indicate that, by 1–2 years of age, γδ T cells have a memory phenotype and the frequencies of Vδ1, Vδ2, and Vγ9 cells are similar to adults [29,30,31,32]. These studied also reported significantly lower frequencies of CD27^+^CD28^+^ (undifferentiated) cells compared to cord blood. Our findings further elucidate the progressive changes in γδ T cells during the first year of life, and the potent effect of CMV infection on these changes. The existence of a public Vδ1 TCR clonotype upon congenital infection in a Belgian cohort [20] was confirmed in the present study. We further identified the presence of this clonotype in a 3/11 infants postnatally infected with CMV at a very early age (<120 days). However, we found that the levels of the public clonotype decayed rapidly and were not detectable beyond 4 months of life despite sustained high viral shedding, providing additional evidence for age-related fetal generation. Notably, several other groups were unable to detect this clonotype in Vδ1 cells of CMV-infected adults [19,23]. It is likely that these observations are due to the specific production of this clonotype in the fetal thymus [25]. Thus, it appears that the remnants of this fetal-like γδ TCR repertoire persist in very young infants and can be induced by CMV infection that occurs in the first few months of life. Additional work is required to understand the kinetics of age and the decay of this public γδ TCR clonotype as we cannot rule out the possibility that this clonotype is present in CMV-uninfected infants and below the level of detection, and in fact we assume that every newborn indeed possesses this clonotype and it is simply expanded with CMV infection at a young age. Furthermore, given that cells expressing this germline-encoded Vγ8Vδ1 clonotype produce IFN-γ responses to CMV and kill CMV-infected cells in vitro [20], studies to ascertain their clinical significance are indicated.

We found that the kinetics of adaptive-like γδ T cell subsets clearly differ from the innate-like Vγ9Vδ2 subset in CMV-infected infants. The frequencies of both Vδ1 and Vδ3 subsets followed a Gaussian trajectory reminiscent of the expansion of αβ T cell responses to primary CMV infection and their subsequent contraction as control of viral replication is gradually achieved [45,46,47]. Furthermore, we found the adaptive-like Vδ1 and Vδ3 subsets from our CMV-infected subjects were phenotypically different than the innate-like Vδ2 compartment. Activation markers examined using spectral flow cytometry suggest that these CMV-associated γδ T cells are cytotoxic to virally infected cells. We discovered that NKG2C expression was almost exclusively in the Vδ1 and Vδ3 subsets. In humans, NKG2C is an activating receptor that recognizes CMV peptides presented by the MHC I-like molecule HLA-E [48,49,50], and healthy CMV-seropositive people have significantly higher frequencies of NKG2C^+^ NK cells, while these cells are rare in CMV-uninfected subjects. NKG2C expression has been described on total γδ T cells [20,51] and on Vδ1 cells, but not previously on Vδ3 cells or in association with CMV infection [52]. Given that CMV infection is uniquely associated with NKG2C expression on NK cells with an adaptive-like phenotype [39,41,53,54], we propose that a similar phenomenon occurs with NKG2C in the γδ T cell immune response to CMV infection.

Similar observations were made on other γ- and δ- paired TCR subsets. Frequencies of the innate-like Vγ9Vδ2 subset were decreased in both CMV-infected infants and adults, while the frequencies of all other TCR pairings were (to varying degrees) increased in CMV-infected infants and adults (compared to age matched uninfected controls). Moreover, frequencies of most adaptive-like paired γδ subsets from CMV-infected subjects plotted versus time had the same Gaussian shape seen in the unpaired Vδ1 or Vδ3 data that we believe is suggestive of a clonal adaptive-like expansion and contraction. Of special interest recently are Vγ9^neg^Vδ2 γδ T cells that were shown to be expanded in CMV-infected fetuses [20]. Our data show that expansion of the Vγ9^neg^Vδ2 subset is especially prominent when CMV infection occurs in the first year of life, and highlights age-specific γδ T cell responses towards CMV given that adults have very minimal to absent frequencies of this subset.

There were several other CMV-associated paired γδ T cell subsets found in both adults and infants with differentiated T effector phenotypes suggestive of adaptive immunobiology. All non-Vγ9Vδ2 γ- and δ- paired TCR subsets developed a unique CMV-associated cluster with similar changes in phenotypic markers. These clusters lost expression of CD27, an indication of a transition from a naïve to late differentiation [17,18,23], and they gained expression of CD16, NKG2C, CD57, HLA-DR, and granzyme A, which are all suggestive of functional anti-viral activity [23,33]. Surface expression of CX3CR1, a chemokine receptor that mediates tissue homing [55], was also increased in each non-Vγ9Vδ2 subsets in CMV-infected individuals. Increased surface expression of CD57, a marker of terminal differentiation, was also detected on all CMV-associated subsets and is suggestive of an adaptive-like memory response. Furthermore, consistent with our findings comparing unpaired Vδ1, Vδ2 and Vδ3 T cell subsets, we did not detect any NKG2C expression on innate-like Vγ9Vδ2 T cells, irrespective of CMV status. The majority of NKG2C was on Vδ1 cells of any pairing and Vγ8/9^neg^Vδ3 cells. Lastly, we found increased CD8α expression on all the CMV-induced adaptive-like subsets and observed an opposite trend in the innate-like Vγ9Vδ2 subset (no difference between CMV-infected and CMV-uninfected subjects). CD8α^+^ γδ T cell expansions have been described in transplant recipients receiving grafts from CMV-infected donors, CMV-infected fetuses [34,36], inflammatory bowel disease [35], in the context of aging (senescence) and CMV infection [56]. NK cells that express CD8 have enhanced killing capacity [57], and expression of CD8α on myeloid cells has been shown to amplify FcR-mediated killing [58]. It is conceivable that the increased CD8α expression we detected on γδ T cells enhances CD16 (FcR)-mediated killing of CMV-infected cells. Collectively, these data are suggestive of an adaptive-like γδ T cell-mediated immune mechanism that might play an important role in the host response against CMV in early life.

As noted above, NKG2C expression was only detected in the adaptive-like and CMV-expanded non-Vδ2 compartment. Furthermore, NKG2C^+^CD57^+^ γδ T cell frequencies were associated with CMV oral shedding, suggesting that these cells may contribute to the control of CMV infection in a similar fashion to NKG2C^+^CD57^+^ NK cells [38,39]. The observational nature of our study precludes the ability to determine a causal effect of these NKG2C^+^CD57^+^ γδ T cells on CMV replication. However, the viral ligand for NKG2C on adaptive NK cells was recently identified to be CMV UL40 peptides presented on HLA-E [50]. Thus, UL40 could also be the viral ligand recognized by NKG2C^+^ γδ T cells that expand during CMV infection. Additional studies are needed to further define the functional importance and mechanism of action of NKG2C^+^ γδ T cells during CMV infection, particularly in early life.

Primary CMV infection in infants is usually clinically asymptomatic [26], and therefore understudied. Expensive prospective cohorts with extensive sampling are needed to capture primary infections, and the result is often small sample sizes and imperfect age-matched controls. A major limitation of this study was the small sample size and a lack of race and environment-matched controls, as virtually all Ugandans are infected with CMV before adulthood and no Ugandan fetal nor cord samples were collected. Although this restricted our ability to exclude impacts of race and environment on γδ T cell frequencies following CMV infection, we were still able to discern important changes in these cells by age and acquisition of primary CMV infection in early life.

## 5. Conclusions

Our data are an important contribution to the growing body of evidence that support critical role for γδ T cells in the human immune response to CMV, particularly in very early post-natal life. Our data are the first to track changes in infant γδ T cells frequency and phenotype during primary CMV infection, of particular importance since young children shedding CMV are a common source of infection for pregnant women. Further evidence indicating that CMV shapes the early γδ T cell repertoire was the striking expansion of Vγ9^neg^Vδ2 cells we found in the majority of infants that acquired CMV in the first year of life and the detection of a previously reported CMV-associated public Vδ1 TCR clonotype in 3 of 11 infants infected with CMV < 120 days old. We report the novel finding that CMV-associated NKG2C^+^ γδ T cells co-express the activation marker CD57 associated with CMV shedding during infancy, and may therefore contribute to the control of infection. Our data have highlighted age-dependent differences in γδ T cells in the context of CMV infection, providing information of potential value for the design of novel clinical therapies for cCMV and/or CMV vaccine design.

## Figures and Tables

**Table 1:** Phenotypic differences of γδ T cell δ-subsets between CMV-infected and CMV-uninfected infants and adults. Expression levels of phenotypic activation markers on δ-subsets obtained by spectral flow cytometry from CMV-infected subjects were compared to CMV-uninfected age controls. Infant subjects were from Ugandan birth cohort (2–12 months old; *n* = 15; CMV-infected *n* = 8 and CMV-uninfected *n* = 7) and adult subjects were Canadian (*n* = 10; CMV-infected *n* = 5 and CMV-uninfected *n* = 5) and Ugandan mothers (*n* = 11; all CMV-infected). Statistical differences between CMV-infected and -uninfected infants and adults were determined using a Wilcoxon–Mann–Whitney test.

		CMV-Infected Infants	CMV-Infected Adults
		**Expression levels compared to uninfected age controls**	
CD16	Vδ1	Higher (***)	Higher (*)
	Vδ2	Higher (*)	Lower (*)
	Vδ3	Higher (***)	Higher (*)
NKG2C	Vδ1	Higher (*)	Higher (*)
	Vδ2	Not detected	Not detected
	Vδ3	Higher (*)	No difference
CD57	Vδ1	Higher (***)	No difference
	Vδ2	No difference	No difference
	Vδ3	Higher (*)	Higher (*)
CD8	Vδ1	Higher (*)	Higher (*)
	Vδ2	No difference	Lower (*)
	Vδ3	Higher (*)	Higher (*)
Granzyme A	Vδ1	Higher (***)	Higher (*)
	Vδ2	No difference	No difference
	Vδ3	Higher (***)	Higher (***)
HLA-DR	Vδ1	Higher (***)	Higher (*)
	Vδ2	Lower (*)	No difference
	Vδ3	Higher (***)	Higher (***)
CX3CR1	Vδ1	Higher (***)	No difference
	Vδ2	No difference	Lower (*)
	Vδ3	Higher (***)	Higher (***)
NKG2C+ CD57+	Vδ1	Higher (*)	Higher (*)
		Not detected in CMV uninfected individuals	
	Vδ2	Not detected	Not detected
	Vδ3	No difference	Higher (***)
		Not detected in CMV uninfected individuals	

* *p* < 0.05, *** *p* < 0.001.

## Supplementary Materials

The following are available online at https://www.mdpi.com/article/10.3390/v13101987/s1, Figure S1: γδ T cell frequencies and phenotypic markers of activation increase with age and EBV infection, Figure S2: γδ T Cell γ-subsets Change with Age and CMV Status, Figure S3: NKG2C^+^CD57^+^ and NKG2C^+^ NK cell frequencies grouped by *NKG2C* genotype.
