# Supplementary material for: Characterization of Adaptive-like γδ T Cells in Ugandan Infants during Primary Cytomegalovirus Infection
Source: Viruses. 2021 Oct 3;13(10):1987. doi: 10.3390/v13101987 (PMC8537190; doi:10.3390/v13101987)
Supplement: Supplementary file 1 [file viruses-13-01987-s001.zip › viruses-1314484-supplementary.pdf]

**Supplementary Materials:**

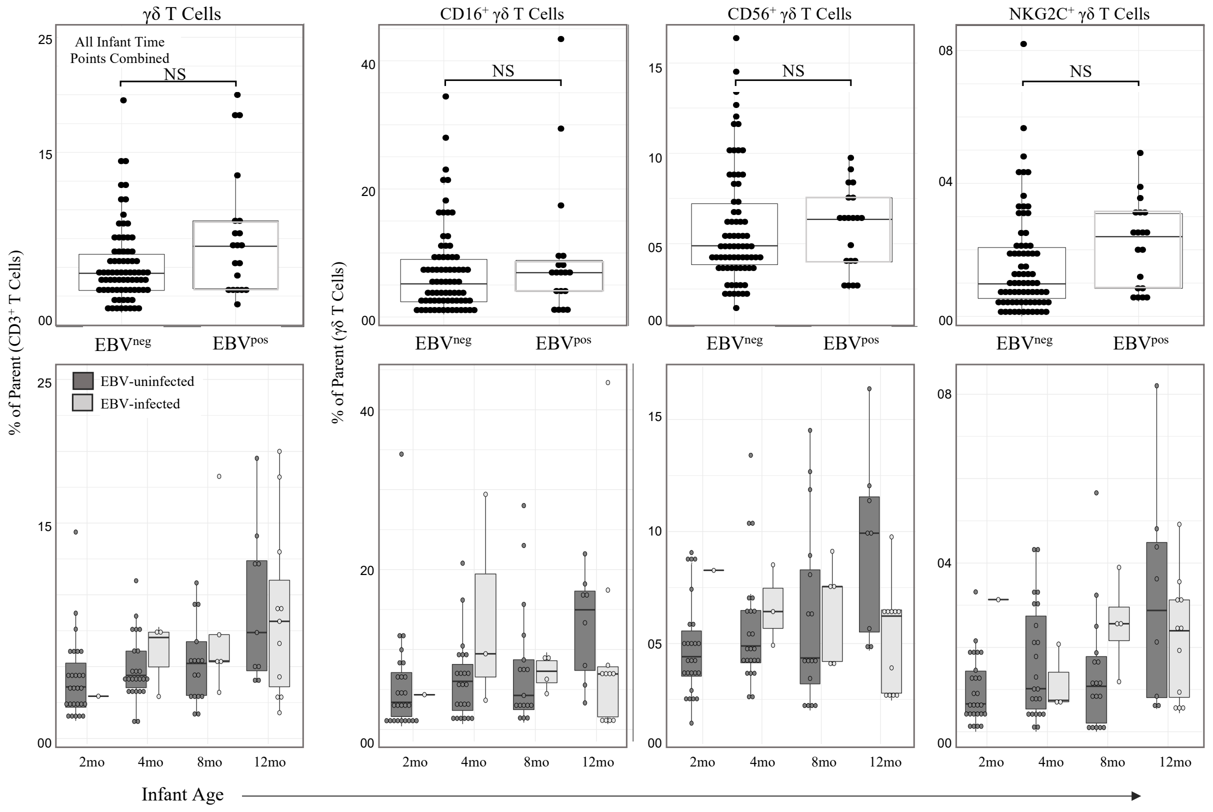


**Figure S1. γδ T cell frequencies and phenotypic markers of activation increase with age and EBV infection.** Total γδ T cells, CD16+, CD56+ and NKG2C+ γδ T cells from EBV-infected (n = 68) and EBV-uninfected infants (n = 19). Top rows depict cell frequencies in longitudinal samples (all time points) and the bottom rows represent the same data plotted by both infection and age in months. A mixed methods linear regression model accounting for repeated measures and testing for age, sex and HIV exposure *in utero* was used to determine statistical associations. Horizontal bars represent the median; boxes extend to the 25th and 75th percentile and whiskers represent the 95th percentiles. P-values were adjusted for multiple comparisons (each cell population tested).

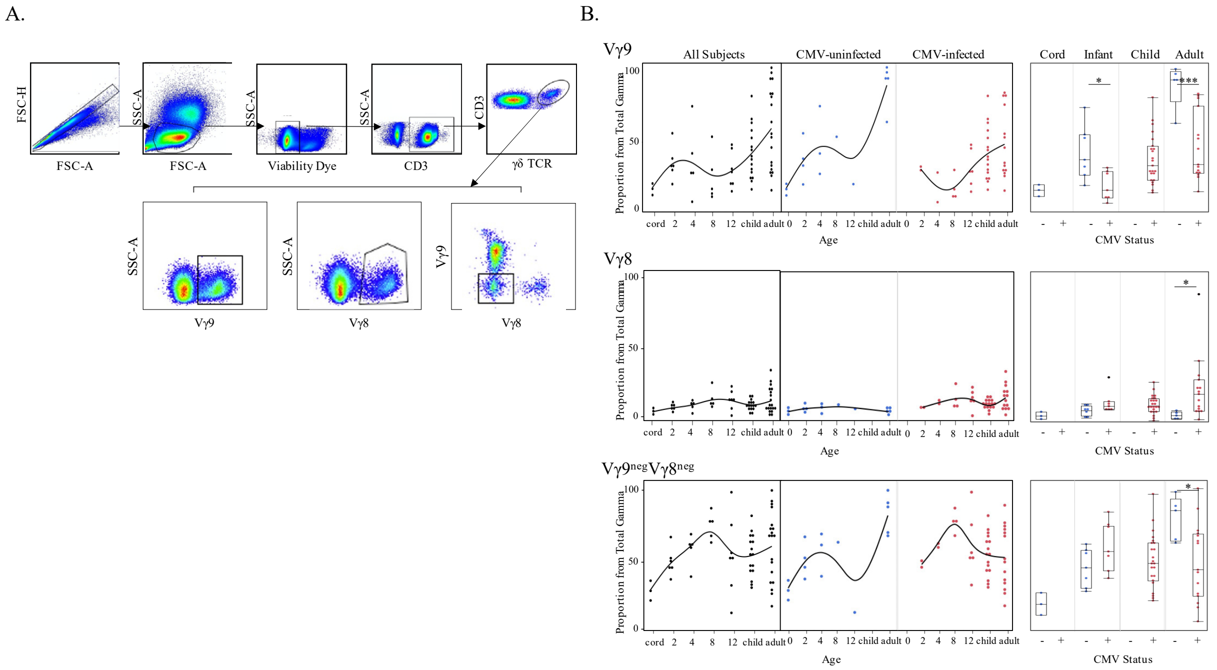


**Figure S2. γδ T Cell γ-subsets Change with Age and CMV Status.** (**A**) Gating strategy for γ-subsets and phenotypes of γδ T cells using spectral flow cytometry. Initial flowCut cleaning in FlowJo was followed by singlet, lymphocyte, $CD3^{+}$ and total γδ T cell gates. Next γ-subsets were gated as shown; (**B**) γ-subset frequencies (proportion of total γδ T cells) were plotted by age (left) and then additionally stratified by CMV status (center). These same data are shown on the right separated by CMV status and grouped by cord blood from Canadian births (n = 3), Ugandan infants (2-12 months old; n = 15; CMV-infected n = 8 and CMV-uninfected n = 7), Ugandan children (1-8 years old; n = 22) and adult subjects were Canadian (n = 10; CMV-infected n = 5 and CMV-uninfected n = 5) and Ugandan mothers (n = 11; all CMV-infected). No cord blood from congenital infections or blood samples from CMV-uninfected children older than 12 months were available for testing with this flow cytometry panel.

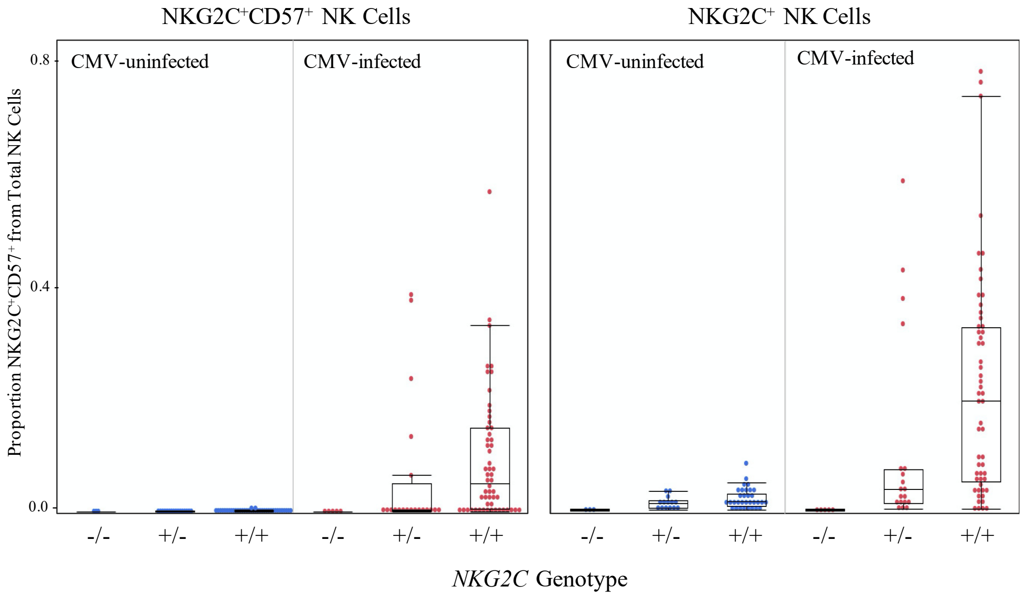


**Figure S3. NKG2C⁺CD57⁺ and NKG2C⁺ NK cell frequencies grouped by *NKG2C* genotype.** Proportions of NKG2C⁺CD57⁺ and NKG2C⁺ NK cells (CD56$^{dim}$CD16⁺ and acquired via conventional flow cytometry) from the Ugandan cohort (all ages) were grouped by *NKG2C* genotype and CMV status. Each data point represents one sample (*NKG2C*-/- n = 8, *NKG2C*-/+ n = 38, *NKG2C*+/+ n = 90).
